# Supplementary material for: Putative Wound Healing Induction Functions of Exosomes Isolated from IMMUNEPOTENT CRP
Source: Int J Mol Sci. 2023 May 18;24(10):8971. doi: 10.3390/ijms24108971 (PMC10218804; doi:10.3390/ijms24108971)
Supplement: Supplementary file 1 [file ijms-24-08971-s001.zip › ijms-2316405-supplementary.pdf]

Supplementary materials

Table S1.- Size distribution of exosomes characterized by AFM and measure through Gwyddion software.

Number	Δx [nm]	Δy [nm]	φ [grad]	R [nm]	Δz [nm]
1	342,42275194	-77,82337694	12,80426992	351,15497867	104,44760883
2	295,72874031	-93,38805233	17,52557350	310,12387229	93,06427895
3	342,42275194	-31,12935078	5,19443052	343,83481139	98,19285477
4	280,16406977	-62,25870155	12,52881149	286,99834827	32,88474158
5	249,03472868	124,51740310	-26,56505831	278,42930838	21,10571851
6	202,34071705	-202,34078004	45,00000892	286,15303082	52,20132329
7	280,16406977	46,69402616	-9,46232510	284,02858671	50,65672896
8	171,21137597	0,00000000	0,00000000	171,21137597	26,43814493
9	217,90538760	-93,38805233	23,19859697	237,07401009	42,93491161
10	249,03472868	-15,56467539	3,57633549	249,52065087	39,54917697
11	186,77604651	-77,82337694	22,61987128	202,34072637	37,01484893
12	295,72874031	0,00000000	0,00000000	295,72874031	30,09493997
13	233,47005814	0,00000000	0,00000000	233,47005814	41,16620237
14	186,77604651	-15,56467539	4,76364317	187,42345283	0,44483727
15	233,47005814	-46,69402616	11,30993590	238,09367931	19,68284588
16	311,29341085	-46,69402616	8,53076823	314,77598339	48,81685503
17	202,34071705	-140,08207849	34,69516188	246,09907454	51,13958549
18	186,77604651	-46,69402616	14,03624766	192,52434555	1,14809022
19	217,90538760	-15,56467539	4,08561805	218,46056180	46,21561559
20	233,47005814	0,00000000	0,00000000	233,47005814	44,69041143
21	186,77604651	0,00000000	0,00000000	186,77604651	48,01030147
22	249,03472868	0,00000000	0,00000000	249,03472868	42,98781055
23	186,77604651	15,56467539	-4,76364317	187,42345283	56,22945115
24	171,21137597	0,00000000	0,00000000	171,21137597	29,23916239
25	217,90538760	-140,08207849	32,73523438	259,04776906	36,61058419
26	311,29341085	-155,64675388	26,56505831	348,03663547	44,80986230
27	202,34071705	-46,69402616	12,99462070	207,65860892	42,39653617
28	202,34071705	-15,56467539	4,39870672	202,93847565	29,69913286
29	155,64670543	-62,25870155	21,80141564	167,63663928	33,16828594
30	186,77604651	-46,69402616	14,03624766	192,52434555	29,22560783

Table S2.- Cellular component present in peptides from the ICRP

Description	P-value	FDR q-value	Enrichment (N, B, n, b)
extracellular region part	4.36E-07	0.000469	1.11 (1283,519,973,435)
extracellular organelle	0.0000133	0.00715	1.10 (1283,475,982,399)
extracellular vesicle	0.0000133	0.00476	1.10 (1283,475,982,399)
extracellular exosome	0.0000133	0.00357	1.10 (1283,475,982,399)
cytosol	0.000046	0.0099	1.08 (1283,735,879,542)
vesicle	0.000752	0.135	1.07 (1283,547,973,445)
keratin filament	0.000972	0.149	1.62 (1283,23,757,22)

Table S3.- Exosome's peptides tissue specificity (abundance ratio 1-100%) according to METASCAPE.

GO	Description	Count	%	Log10(P)	Log10(q)
PGB:00017	Tissue-specific: skin	32	4.00	-15.00	-12.00
PGB:00156	Tissue-specific: Tongue	12	1.50	-10.00	-8.10
PGB:00081	Cell-specific: Bronchial Epithelial Cells	20	2.50	-9.20	-7.10
PGB:00041	Tissue-specific: Blood	20	2.50	-5.20	-3.60
PGB:00078	Cell-specific: liver cell	10	1.20	-3.80	-2.40
PGB:00005	Tissue-specific: tonsil	8	1.00	-3.80	-2.30
PGB:00103	Tissue-specific: Bladder	5	0.62	-3.40	-2.00
PGB:00060	Tissue-specific: retinoblastoma	9	1.10	-3.00	-1.70
PGB:00001	Tissue-specific: liver	31	3.90	-2.50	-1.30
PGB:00010	Tissue-specific: adipose tissue	11	1.40	-2.20	-1.00
PGB:00059	Cell-specific: Testis Germ Cell	7	0.88	-2.10	-0.98

Table S4.- Peptides from exosomes interact with component of the PIP3-AKT pathway.

1.- Interactors of PI (4,5) P2	Reactome score	6.- Interactors of MKRN1	Reactome score	15.- Interactors of: p-T23-CHUK	Reactome score
TLN1	0.59	SDCBP2	0.556	CDC37	0.744
CDC42	0.564	NAA10	0.556	HSP90AA1	0.609
EPB41L2	0.462	RAD23A	0.556	PYCARD	0.512
SNX2	0.462	RAD23B	0.556	16.- Interactors of: p-S196-S188-MDM2	Reactome score
FLNB	0.462	BYSL	0.556	USP7	0.994
SRP72	0.462	7.- Interactors of: AKT1S1	Reactome score	RPL11	0.932
ACTN1	0.462	YWHAB	0.527	NCL	0.71
RAC2	0.462	8.- Interactors of: TSC2	Reactome score	EEF1A1	0.709
EZR	0.462	YWHAB	0.93	NPM1	0.697
MX1	0.462	YWHAZ	0.903	RPS3	0.686
FLNA	0.462	YWHAG	0.724	OTUB1	0.632
CFL1	0.462	YWHAQ	0.563	PML	0.609
MSN	0.462	PPP1CA	0.563	RPS27	0.602
KIF5B	0.462	9.- Interactors of: CHUK	Reactome score	HNRNPK	0.589
MYH9	0.462	CDC37	0.744	RPL23	0.573
IQGAP1	0.462	HSP90AA1	0.564	RPS20	0.527
DNM2	0.462	PYCARD	0.512	EZR	0.524
RAB7A	0.462	10.- Interactors of: MDM2	Reactome score	FKBP3	0.508
CAPZA1	0.462	USP7	0.959	17.- Interactors of: CREB	Reactome score

MYL6	0.462	RPL11	0.912	HTT	0.556
ACTB	0.462	NCL	0.71	18.- Interactors of: NR4A1	Reactome score
RACK1	0.462	EEF1A1	0.709	PRDX4	0.533
CLTC	0.462	NPM1	0.697	VASP	0.524
SPTBN1	0.462	RPS3	0.686	19.- Interactors of: p-S133-CREB	Reactome score
ILF3	0.462	OTUB1	0.632	HTT	0.556
TRIM28	0.462	PML	0.582	20.- Interactors of: p-S351-NR4A1	Reactome score
SPTAN1	0.462	RPL23	0.573	PRDX4	0.553
FLNC	0.462	S100A16	0.558	VASP	0.524
KPNB1	0.462	RPS20	0.527		
SEPTIN2	0.462	EZR	0.524		
SEPTIN7	0.462	FKBP3	0.508		
BZW1	0.462	11.- Interactors of: p-S183, T246-AKT1S1	Reactome score		
MYH14	0.462	YWHAB	0.527		
CAND1	0.462	12.- Interactos of: p-S109-MKRN1	Reactome score		
2.- Interactors of: PI (3,4,5) P3	Reactome score	SDCBP2	0.556		
ITGB1	0.462	NAA10	0.556		
3.- Interactors of: PDK1	Reactome score	RAD23A	0.556		
YWHAQ	0.544	RAD23B	0.556		
CSK	0.488	BY5L	0.556		
4.- Interactors of: PTEN	Reactome score	13.- Interactors of: p-S99-BAD	Reactome score		
PRDX1	0.59	YWHAZ	0.851		
PPP1CA	0.524	SFN	0.804		
CAV1	0.499	YWHAQ	0.687		
DBN1	0.471	YWHAE	0.667		
MX1	0.462	YWHAB	0.659		
5.- Interactors of: BAD	Reactome score	YWHAG	0.659		
YWHAZ	0.851	CAPN1	0.556		
SFN	0.804	14.- Interactors of: p-S939, T1462-TSC2	Reactome score		
YWHAQ	0.687	YWHAB	0.903		
YWHAE	0.667	YWHAZ	0.903		
YWHAB	0.93	YWHAG	0.724		
YWHAG	0.659	YWHAQ	0.702		
CAPN1	0.556	PPP1CA	0.544		

Table S5.- Peptides from exosomes interact with component of the cell-cell junction signaling pathway.

1.- Interactors of: CRB3	Reactome score	CCT4	0.643	15.- Interactors of: ARHGEF6	Reactome score
PSMA3	0.556	HSPB1	0.564	PAK2	0.709
2.- Interactors of: MPP5	Reactome score	LIMS1	0.556	GIT2	0.631
GSN	0.556	ATP2A2	0.527	KRT27	0.556
HTT	0.556	SEC61A1	0.527	NAA10	0.524
3.- Interactors of: PVR	Reactome score	CCT2	0.527	16.- Interactors of: ACTN1	Reactome score
PLP2	0.556	TIMM50	0.527	TXN	0.556
AFDN	0.646	GCN1	0.527	ACTN1	0.553
FLNA	0.556	TECR	0.527	PDLIM7	0.527
4.- Interactors of: PVRL1	Reactome score	9.- Interactors of: PXN	Reactome score	17.- Interactors of COL17A1 (1-1497)	Reactome score
SDCBP	0.650	PTK2	0.91	UBQLN2	0.556
5.- Interactors of: CTNNA1	Reactome score	GIT2	0.688		
CTNNA1	0.618	CSK	0.623		
VCL	0.558	SORBS1	0.61		
JUP	0.519	GIT1	0.564		
6.- Interactors of: ANG	Reactome score	ITGB1	0.524		
ANXA2	0.484	FN1	0.491		
S100A10	0.484	PTK2	0.491		
7.- Interactors of: ITGB1	Reactome score	10.- Interactors of: PARVA	Reactome score		
FLNA	0.819	LIMS1	0.527		
FN1	0.791	11.- Interactors of: TESK1	Reactome score		
TLN1	0.571	YWHAB	0.611		
EGLN1	0.524	12.- Interactors of: PLEC	Reactome score		
FABP3	0.471	HTT	0.666		
8.- Interactors of: ILK	Reactome score	WNK1	0.534		
LIMS1	0.952	FUS	0.518		
PARVG	0.831	13.- Interactors of: RSU	Reactome score		
CCT5	0.643	LIMS1	0.801		
CCT3	0.643	14.- Interactors of: VASP	Reactome score		

CCT8	0.643	ZYX	0.666

Table S6.- Peptides from exosomes interact with component of the FGFR1 signaling pathway

1.- Interactors of: p-4Y-PLCG1	Reactome score	FLNB	0.462	YWHAZ	0.527
KHDRBS1	0.692	SRP72	0.462	YWHAQ	0.758
RIN3	0.602	ACTN1	0.462	YWHAB	0.643
LCP2	0.564	RAC2	0.462	YWHAG	0.527
CAV1	0.525	EZR	0.462	YWHAE	0.527
2.- Interactors of PLCG1	Reactome score	ANXA7	0.462	10.- Interactors of: BRAF	Reactome score
KHDRBS1	0.692	MX1	0.462	MAP2K1	0.900
RIN3	0.602	FLNA	0.462	YWHAZ	0.899
LCP2	0.564	CFL1	0.462	YWHAB	0.818
CAV1	0.525	MSN	0.462	YWHAQ	0.813
3.- Interactors of: PI (3,4,5) P5	Reactome score	KIF5B	0.462	YWHAG	0.813
ITGB1	0.462	MYH9	0.462	HSP90AB1	0.669
RAC2	0.462	IQGAP1	0.462	YWHAE	0.643
IQGAP1	0.462	DNM2	0.462	SFN	0.564
CAPZB	0.462	RAB7A	0.462	IQGAP1	0.544
DNM2	0.462	CAPZA1	0.462	HSPA5	0.527
AP2B1	0.462	MYL6	0.462	HSPA8	0.527
CLTC	0.462	ACTB	0.462	HSPA9	0.527
SPTBN1	0.462	RACK1	0.462	CDC37	0.527
SPTAN1	0.462	CLTC	0.462	11.- Interactors of: p-S111, S120-SPRY2	Reactome score
EHD1	0.462	SPTBN1	0.462	GRN	0.556
4.- Interactors of: FRS3	Reactome score	ILF3	0.462	LASP1	0.556
KRT33B	0.556	TRIM28	0.462	SH3KBP1	0.499
ECM1	0.556	SPTAN1	0.462		
POF1B	0.556	FLNC	0.462		
PDLIM7	0.556	KPNB1	0.462		
5.- Interactors of: PTPN11	Reactome score	SEPTIN2	0.462		
EEF1A1	0.544	SEPTIN7	0.462		
CAV1	0.499	BZW1	0.462		
6.- Interactors of: PIK3CA	Reactome score	MYH14	0.462		
HTT	0.556	CAND1	0.462		
7.- Interactors of: (4,5) P2	Reactome score	8.- Interactors of: p-T250, T255, T385, S437-MKN	Reactome score		

TLN1	0.590	HTT	0.556
CDC42	0.564	9.- Interactors of: CBL	Reactome score
EPB41L2	0.462	SH3KBP1	0.973
SNX2	0.462	HTT	0.556

Table S7.- Peptides from exosomes interact with component of the IGF1R signaling pathway.

Interactors of: IRS2	Reactome score
YWHAB	0.688
YWHAG	0.688
YWHAZ	0.669
YWHAQ	0.564

Table S8.- Peptides from exosomes interact with component of the Sonic Hedgegog signaling pathway.

1.- Interactors of: CDON	Reactome score	KRT16	0.556	PPP2R5E	0.581
ABL1	0.524	KRT15	0.556	TNS2	0.556
2.- Interactors of: ADRBK1	Reactome score	BYSL	0.556	8.-Interactors of: ITCH	Reactome score
GIT1	0.463	LASP1	0.556	SXN6	0.623
3.- Interactors of: CSNK1A1	Reactome score	CDC37	0.556	SPART	0.527
RIPK1	0.602	KRT27	0.556	SH3GL2	0.526
SEC16A	0.527	CPSF7	0.556	9.- Interactors of NUMB	Reactome score
HMGB1	0.488	EIF1AX	0.556	EPS15L1	0.656
HMGB2	0.488	DDX17	0.556	REPS1	0.499
PPP1R14A	0.488	6.- Interactors of: CDC73	Reactome score		
4.- Interactors of: SUFU	Reactome score	LEO1	0.955		
KRT75	0.556	POLR2A	0.708		
KRT27	0.556	KMT2A	0.576		
5.- Interactors of: GAS8	Reactome score	RNF20	0.556		
SORBS3	0.556	7.- Interactors of: DZIP1	Reactome score		
KRT14	0.556	PPP2R5A	0.666		
